# Teneligliptin As an Initial Therapy for Newly Diagnosed, Drug Naive Subjects With Type 2 Diabetes

**DOI:** 10.14740/jocmr1841e

**Published:** 2014-05-22

**Authors:** Eiji Kutoh, Mitsuru Hirate, Yu Ikeno

**Affiliations:** a Biomedical Center, Tokyo, Japan; b Division of Diabetes and Endocrinology, Department of Internal Medicine, Gyoda General Hospital, Saitama, Japan

**Keywords:** Teneligliptin, DPP-4 inhibitor, Insulin resistance, Beta-cell function

## Abstract

**Background:**

Teneligliptin is a novel, highly selective dipeptidyl peptidase-4 (DPP-4) inhibitor. The aim of this study is to explore the glycemic and non-glycemic efficacies of teneligliptin as an initial therapy.

**Methods:**

Newly diagnosed, drug naive Japanese subjects with type 2 diabetes (T2DM) were assigned to 20 mg/day teneligliptin monotherapy (n = 31). At 3 months, levels of glycemic and other parameters were compared with those at baseline.

**Results:**

Significant reductions of HbA1c (from 10.34 ± 2.06 to 8.38 ± 2.23%) and fasting blood glucose (FGB, from 211.3 ± 68.4 to 167.3 ± 70.2 mg/dL) levels were observed without any clinically significant adverse events. However, significant increases of uric acids (UA) levels were observed and two subjects reported mild hypoglycemic events. Homeostasis model assessment-B (HOMA-B) levels significantly increased, while high HOMA-R levels significantly decreased. Significant correlations were observed between the changes (Δ) of HbA1c and those of HOMA-B, and between ΔFBG and ΔHOMA-R. No changes in lipid and body weight were noted.

**Conclusions:**

Teneligliptin might be effectively and safely used as an initial therapy for newly diagnosed T2DM. Glycemic efficacy of teneligliptin is obtained through activating beta-cell function as well as decreasing insulin resistance.

## Introduction

With an increasing number of newly diagnosed patients with T2DM worldwide, it is important to establish therapeutic strategies for those patients. Currently metformin together with life style modifications (healthy eating, body weight control, increased physical activity) is regarded as the initial drug to start [1], although other drugs could be potential candidates as well. For example, in patients with renal or heart failure where metformin is contraindicated, and/or in elderly individuals or those with corticosteroid-induced diabetes, the use of other drugs as the first-line therapy may be justifiable and reasonable [2, 3]. Dipeptidyl peptidase-4 (DPP-4) inhibitors have recently emerged as a new class of oral hypoglycemic agent and show favorable results in improving glycemic control (in particular postprandial hyperglycemic control) with low risk of hypoglycemia and weight gain, and overall good tolerability profile [4-6]. DPP-4 inhibitors are associated with enhanced beta-cell function, making them a good therapeutic option early in the disease when the patients still maintain sufficient levels of beta-cell function [7-9].

Teneligliptin, a novel chemotype prolylthiazolidine-based DPP-4 inhibitor, shows a unique chemical structure which is characterized by five consecutive rings (J-shaped), thereby potentially producing unique characteristics including its glucose lowering efficacy and half-time [10-12]. It is administered with 20 - 40 mg once daily. Since the metabolites of this drug are excreted through hepatic (approximately 35%) and renal (about 65%) route, no dose adjustment is necessary in patients with renal impairment [13, 14]. The efficacy and safety profiles of teneligliptin are similar to those of other DPP-4 inhibitors [15, 16]. Particularly because of its long half-life (approximately 24 h [10, 14]), this drug was shown to stabilize the glucose fluctuations throughout the day [15, 17].

Since teneligliptin is currently marketed only in Japan, limited data and information are available in actual clinical settings. Furthermore, it is not at all clear whether or not teneligliptin is appropriate for the initial drug for patients with T2DM. Thus it is of therapeutic value to analyze the glycemic and non-glycemic efficacies of teneligliptin under such circumstances. To undertake such studies, it makes sense to perform with drug naive subjects as monotherapy in order to eliminate the influences of other drugs as much as possible. As an initial step towards investigating these issues, teneligliptin 20 mg/day monotherapy was performed with newly diagnosed, drug naive subjects with T2DM and effects on a number of glycemic and non-glycemic parameters were investigated.

## Subjects and Methods

### Subjects

A project of monitoring the effects of oral hypoglycemic drugs in newly diagnosed, drug naive Japanese subjects with 2TDM is ongoing in our group. Inclusion criteria were those who had been recently diagnosed with T2DM according to the criteria of the Japan Diabetes Society [18] and had not received any regularly prescribed drugs in the 3 months prior to the study. The work described in this manuscript is part of this project and its aim is to study the glycemic and non-glycemic efficacies of teneligliptin in newly diagnosed, drug naive Japanese subjects with T2DM. Exclusion criteria were those with clinically significant renal creatinine (CRE) > 1.5 mg/dL, liver glutamic oxalacetic transaminases/glutamic pyruvic transaminases (GOT/GPT) > 70/70 IU/L), hypertensive (blood pressure above 160/100 mm Hg) disorders, type 1 diabetes (T1DM) and pregnancy. These subjects were recruited from the outpatient Division of Diabetes and Endocrinology, in Department of Internal Medicine, Gyoda General Hospital (Saitama, Japan). Most of these patients were identified by the health check screening system usually performed twice a year in Japan. These patients received 20 mg/day teneligliptin monotherapy (n = 31). These subjects were encouraged to follow the exercise suggested by the American Diabetes Association [19]. Informed consents were obtained from the patients, and the protocol for this study was approved by the Ethical Committee. In the case of unacceptable or undesirable therapeutic outcome, the patients were free to leave therapy whenever they wished.

### Laboratory measurements

The primary end-point was the change of HbA1c levels from baseline to 3 months. The HbA1c values were assessed by the NGSP standardization [20, 21]. The secondary end-points were the changes of fasting blood glucose (FBG) and other metabolic parameters including total cholesterol (T-C), triglyceride (TG), high density lipoprotein cholesterol (HDL-C), low density lipoprotein (LDL-C), free fatty acid (FFA), uric acid (UA), HOMA-R, HOMA-B and BMI.

Blood was collected in the fasting state before breakfast from the cutaneous vein, followed by the analysis at the central clinical laboratory of the Gyoda General Hospital. Measurements of HbA1c and FBG (measured by the system form Arkray, Shiga, Japan) were performed once a month. Insulin (measured by the kit from Abbott Japan, Tokyo), T-C, TG, HDL-C, LDL-C (measured by the kit from Nittobo, Tokyo, using Hitachi 7180 analyzer) and FFA (Mitsubishi BML, Tokyo, Japan) were measured at the start (baseline) and at 3 months of the study. Anti-glutamic acid decarboxylase (GAD) antibody was measured in some suspected patients in order to exclude those with T1DM (Mitsubishi BML, Tokyo, Japan). HOMA-R and HOMA-B were calculated as described [22] HOMA-R = IRI (µU/mL) × FBG (mg/dL)/405, HOMA-B = IRI (µU/mL) × 360/FBG (mg/dL) - 63. Liver (GOT, GPT, ALP and γ-GTP) and renal (BUN and CRE) functions were also monitored monthly. In the case of any significant increase in these parameters, administration of teneligliptin was planned to discontinue. The drop-out subjects were excluded from data analysis.

### Data analyses

Change was calculated as the values at 3 months (post-therapy) minus those at baseline (pre-therapy). Paired Student’s *t*-test was used to analyze the changes in each parameter. Multiple regression analysis was performed to determine the contributing factors to the changes of HbA1c levels. The following independent variables (baseline levels) including age, HbA1c, FBG, HDL-C, TG, LDL-C, UA, HOMA-R, HOMA-B and BMI were used. Simple regression analysis was performed to analyze the changes between measured parameters. The results were expressed as the mean ± SD. Throughout the statistical analysis, values of P < 0.05 were considered significant.

## Results

### Safety and tolerability

Two out of 31 subjects reported mid hypoglycemic events, which could be easily managed by taking glucose drinks by themselves. One subject reported mild constipation and another complained of skin rashes. These potential adverse events occurred in the first 4 weeks of the initiation of the drug. Significant increases of UA levels were observed (Table 1). Otherwise no subjects had any clinically significant elevations of renal or hepatic enzymes and no gastrointestinal complains were observed. No subject had dropped out because of intolerance or adverse events.

**Table 1:** Changes of Glycemic and Non-Glycemic Parameters With Teneligliptin

	Baseline	3 months	P values
Age	58.29 ± 14.95		
HbA1c (%NGSP)	10.34 ± 2.06	8.38 ± 2.23	< 0.00001
FBG (mg/dL)	211.3 ± 68.4	167.3 ± 70.2	< 0.0002
Insulin (μU/mL)	7.68 ± 9.24	7.55 ± 5.15	n.s.
HOMA-R	3.74 ± 4.28	2.90 ± 2.16	n.s.
HOMA-B	24.04 ± 31.14	40.23 ± 40.98	< 0.00001
T-C (mg/dL)	218.3 ± 33.4	217.2 ± 38.9	n.s.
TG (mg/dL)	126.7 ± 46.8	128.0 ± 66.4	n.s.
HDL-C (mg/dL)	56.4 ± 15.4	57.2 ± 15.9	n.s.
Non-HDL-C (mg/dL)	161.8 ± 32.3	159.9 ± 39.0	n.s.
LDL-C (mg/dL)	140.2 ± 30.9	141.1 ± 32.7	n.s.
FFA (mEq/dL)	0.754 ± 0.361	0.676 ± 0.253	n.s.
UA (mg/dL)	4.69 ± 1.41	5.15 ± 1.55	< 0.05
BMI	23.90 ± 4.61	23.90 ± 3.99	n.s.

Paired Student’s *t*-test was used to compare the changes of the indicated parameters before and after treatment. The results are expressed as the mean ± SD.

### Effect of teneligliptin on glycemic control

At 3 months, significant reductions of HbA1c and FBG levels were observed (for each value and statistical significance, Table 1). Ten out of 31 subjects were non-responders whose HbA1c had less than (<) 1% reduction from the baseline. Eleven out of 31 subjects achieved HbA1c < 7%.

**Figure 1:**
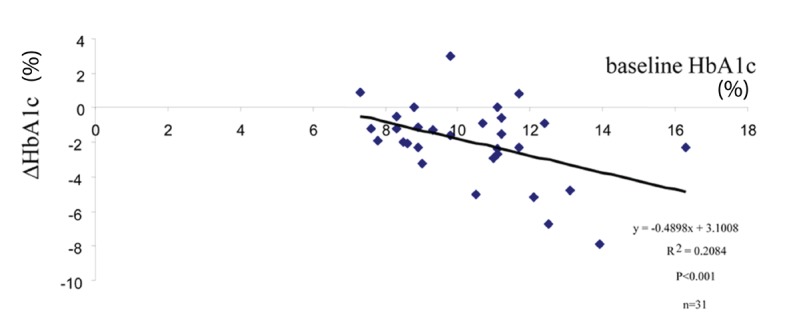
Baseline-dependent glucose lowering efficacy of teneligliptin. Simple regression analysis was performed between the changes of (Δ) HbA1c and baseline HbA1c levels.

In an effort to find any predictive parameters for the response (glycemic efficacy) of teneligliptin, multiple regression analysis was performed between the changes of (Δ)HbA1c levels (as dependable variable) and the baseline levels of metabolic parameters including age, HbA1c, FBG, insulin, HOMA-R, HOMA-B, TG, HDL-C, non-HDL-C, LDL-C, UA and BMI (as independent variables). Among these factors, the baseline HbA1c level was selected as the significant contributing factor for ΔHbA1c (Table 2). This result was confirmed by simple regression analysis showing that significant negative correlations existed between ΔHbA1c and baseline HbA1c levels (Fig. 1).

**Table 2:** Multiple Regression Analysis With the Factors Associated With the Changes of HbA1c With Teneligliptin

	Coefficient	Standard error	t	P	R2
	2.2249	6.7479	0.32972	0.74542	0
Age	-0.018583	0.03449	-0.5388	0.59662	0.0041531
Baseline HbA1c	-0.69467	0.28351	-2.4502	0.024733	0.20841
FBG	0.010769	0.013359	0.80615	0.43068	0.00029303
Insulin	0.35054	0.7305	0.47986	0.63711	0.039977
HOMA-R	-0.79265	1.1315	-0.70054	0.49254	0.039729
HOMA-B	-0.021344	0.084418	-0.25283	0.80326	0.030499
TG	0.02177	0.015404	1.4133	0.17462	0.017047
HDL-C	0.027854	0.043685	0.6376	0.53176	0.026703
Non-HDL-C	-0.047243	0.048644	-0.97121	0.34431	0.037563
LDL-C	0.047284	0.048906	0.96684	0.34644	0.075844
UA	-0.33518	0.40987	-0.81776	0.42419	0.013704
BMI	0.035169	0.20015	0.17571	0.86248	0.023904

Dependent variables: changes of HbA1c levels. Independent variables: age, baseline values of HbA1c, FBG, insulin, HOMA-R, HOMA-B, TG, non-HDL, LDL-C, UA and BMI.

### Effect of teneligliptin on beta-cell function and insulin resistance

**Figure 2:**
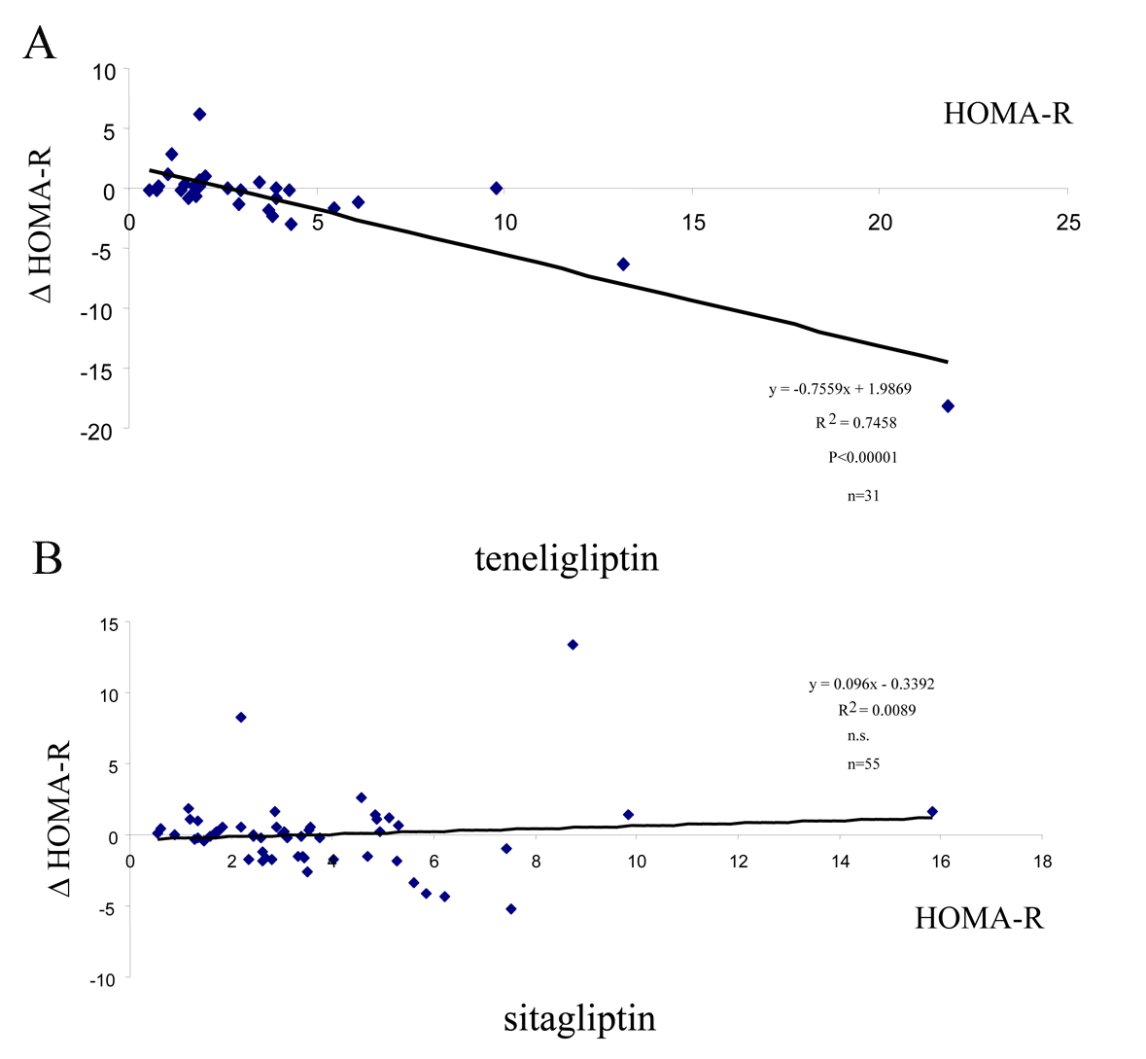
Down-regulation of high HOMA-R levels. Simple regression analysis was performed between the changes of (Δ) HOMA-R and baseline HOMA-R levels. (A) teneligliptin. (B) sitagliptin.

DPP-4 inhibitors are known to augment beta-cell function; however, their effects on insulin resistance (sensitivity) remain uncharacterized. This question has been investigated using HOMA-B (for beta-cell function) and HOMA-R (for insulin resistance) indexes. As shown in Table 1, HOMA-B levels significantly increased, while HOMA-R levels had a tendency to decrease. Then, subjects were divided into two groups; those with high HOMA-R (> 3.5, n = 11) and others with normal-low HOMA-R (< 3.5, n = 20) levels. As indicated in Table 3, high HOMA-R levels significantly decreased with teneligliptin. To confirm this, simple regression analysis without dividing the subjects into two groups was performed between ΔHOMA-R and the baseline HOMA-R levels. As shown in Fig. 2A, significant negative correlations were observed between these parameters. In the past few years, our group was studying the effect of sitagliptin, the most extensively used DPP-4 inhibitor worldwide, on metabolic parameters in newly diagnosed, drug naive patients. With sitagliptin, no such observations were identified (n = 55, Fig. 2B).

**Table 3:** Effects on Insulin Sensitivity (HOMA-R)

Baseline	3 months	P values
7.30 ± 5.67	4.08 ± 2.45	< 0.05
1.78 ± 0.74	2.34 ± 1.72	n.s.

Paired Student’s *t*-test was used to compare the changes of HOMA-R values before and after treatment in two groups with high (n = 11) and normal-low (n = 20) HOMA-R levels. The results are expressed as the mean ± SD.

**Figure 3:**
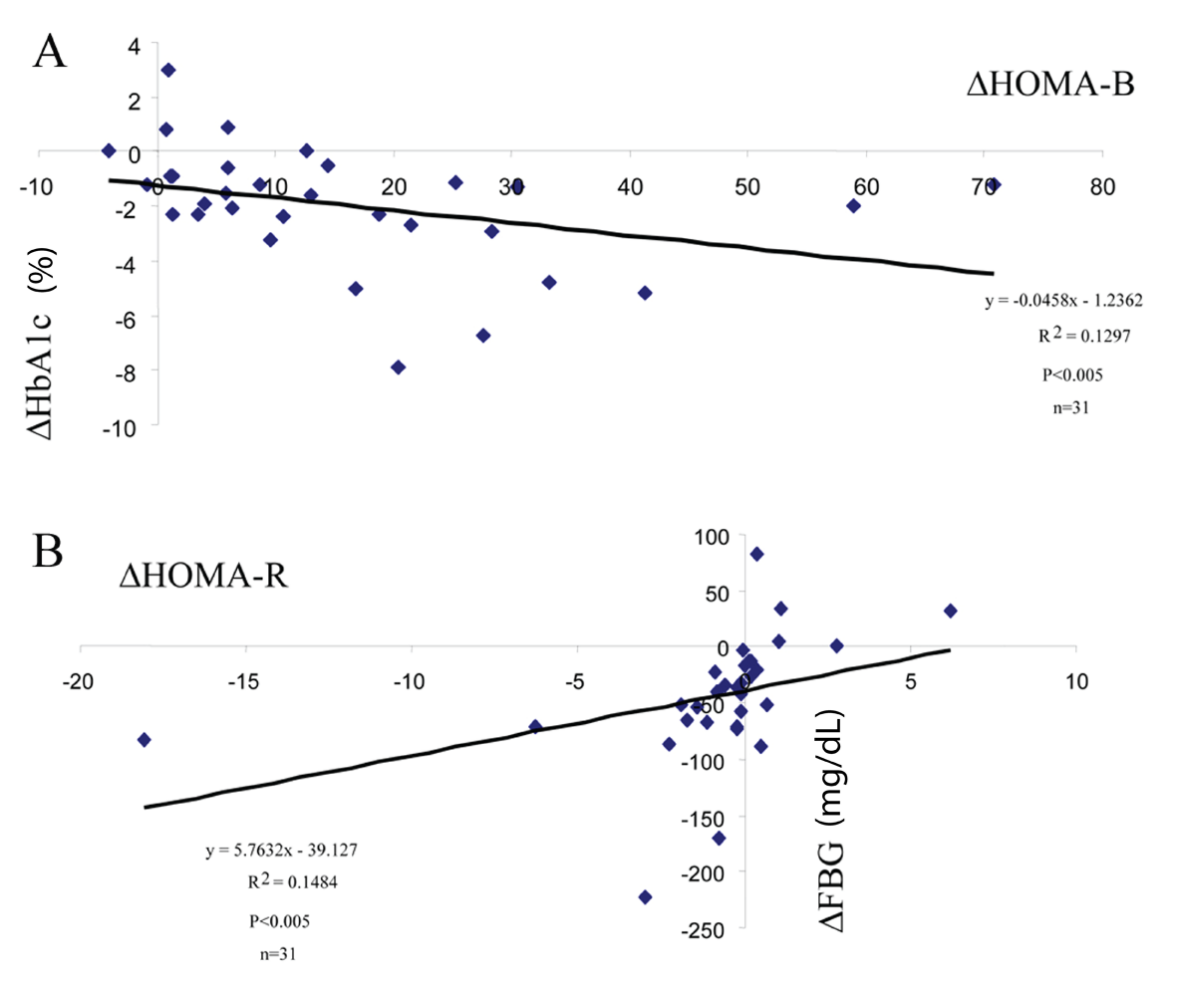
Correlations between glucose lowering efficacy and activation of beta-cell function or relieving insulin resistance. Simple regression analysis was performed between the indicated parameters. (A) ΔHbA1c and ΔHOMA-B. (B) ΔFBG and ΔHOMA-R.

Next, one interesting question is whether the reductions in glucose levels (HbA1c and FBG) have any correlations with the changes of the above mentioned parameter (HOMA-B or HOMA-R indexes). For this purpose, simple regression analysis was performed between these parameters. Significant negative correlations were observed between the changes of (Δ) HbA1c and ΔHOMA-B (Fig. 3A), but not between ΔHbA1c and ΔHOMA-R (results not shown). By contrast, significant correlations were observed between ΔFBG and ΔHOMA-R (Fig. 3B), but not between ΔFBG and ΔHOMA-B (result not shown).

### Effect of teneligliptin on lipid, body weight and blood pressure

Effects on DPP-4 inhibitors on non-glycemic parameters, for example, lipid profiles, are controversial. As an initial step towards investigating the effects of teneligliptin on lipids, a number of lipid parameters including T-C, TG, HDL, non-HDL-C, LDL-C or FFA were monitored. However, no statistically significant changes of these parameters were observed (Table 1). However, FFA had a tendency to decrease (Table 1). No changes in body weight (as measured by body mass index: BMI) were noted (Table 1). Blood pressure was also monitored. The variations were so large and no conclusions have been made regarding the effect of teneligliptin on blood pressure (results not shown).

## Discussion

### Glycemic effects and safety of teneligliptin

In the present work, teneligliptin 20 mg/day monotherapy in newly diagnosed, drug naive Japanese subjects with T2DM was shown to be rather effective in reducing blood glucose levels (both HbA1c and FBG levels, Table 1) without any clinically significant adverse events on kidney or liver. This glucose-lowering efficacy is comparable to other DPP-4 inhibitors tested in the identical environment (sitagliptin or alogliptin in drug naive subjects as monotherapy [9, 23]). In analogy to other OHAs, the response to teneligliptin is proportional to the baseline HbA1c levels (Fig. 1). However, two out of 31 of the subjects reported mild hypoglycemic events. No gastrointestinal complains or body weight changes were noted (Table 1). However, significant elevations of UA levels, although still within normal range, were observed (Table 1). Currently it is unclear whether the elevated levels of UA have any impact on the increased risk for gout or cardiovascular disorders. Although the numbers of the subjects in this study are small and the study duration is short, these results implicate that teneligliptin could be effectively and safely used as one of the first-line drugs for T2DM.

### Effect of teneligliptin on beta-cell function and insulin sensitivity

What are the underlying potential mechanisms for the good glycemic efficacy of teneligliptin? DPP-4 inhibitors are a class of incretin-based drugs that stimulate insulin secretion. It was shown that teneligliptin up-regulated beta-cell function (as estimated by HOMA-B, Table 1) and the changes of (Δ) HOMA-B were negatively correlated with those of HbA1c (Fig. 3A) but not with those of FBG (data not shown).

In comparison to GLP-1 receptor agonists, the effect of DPP-4 inhibitors on beta-cell function is less well characterized. DPP-4 inhibitors appear to improve beta-cell function when measured by HOMA-B and proinsulin/insulin ratio [24-26]. The assessments of DPP-4 inhibitors on beta-cell function require further investigation beyond these parameters. Specifically, it is of significance to study whether DPP-4 inhibitors possess beneficial effects on beta-cell mass, and consequently they can eventually delay or convert the progression of the disease process.

Effects of DPP-4 inhibitors on insulin sensitivity (resistance) could be an interesting research area but are scant at present. In the present study, it was shown that teneligliptin decreased HOMA-R levels in the subjects with high baseline HOMA-R levels (Table 1 and Fig. 2A) and changes of (Δ) HOMA-R levels were correlated with ΔFBG (Fig. 3B), but not with ΔHbA1c (result not shown).

These results indicate that the glycemic effect of teneligliptin is obtained through decreasing insulin resistance as well as activating beta-cell function. However this was not the case with sitagliptin (Fig. 2B). The finding that teneligliptin could ameliorate insulin sensitivity (resistance) may be a unique character of this drug. In order to consolidate the above findings, euglycemic clamp study in humans will be required in order to prove that teneligliptin has indeed beneficial effects on insulin sensitivity.

### Effect of teneligliptin on non-glycemic parameters

Effects of DPP-4 inhibitors on non-glycemic parameters such as lipids are controversial [27]. In general, it is regarded as lipid neutral. Recently our group has shown that alogliptin or sitagliptin has favorable effects on lipid profiles (sitagliptin decreases TG and FFA while alogliptin down-regulates atherogenic cholesterols including LDL-C or non-HDL-C [9, 23]).

In the present study, we tested whether teneligliptin has any effects on lipid profiles. However, little effects, if any, on lipid parameters were noted (Table 1). Although all the DPP-4 inhibitors inhibit the same enzyme (DPP-4) and display similar glucose lowering efficacies, they show clearly distinct chemical structures and pharmacological properties such as half-life, bioavailability, protein binding, metabolism, presence of active metabolites or excretion route [28-30]. These differences may have distinct impact on lipids or other non-glycemic profiles.

### The limitations and strengthens of the study

The limitations of this study are that the number of the subjects is small and the study duration is short. However one can assume that the observed changes were caused exclusively by teneligliptin based on the design of the study (monotherapy with drug naive patients). Further randomized, double-blind, placebo-controlled longer period study with increased number of subjects will be necessary to strengthen the finding in this study.
